# Occlusal Splints and Exercise Performance: A Systematic Review of Current Evidence

**DOI:** 10.3390/ijerph181910338

**Published:** 2021-09-30

**Authors:** Leonardo Cesanelli, Gianfranco Cesaretti, Berta Ylaitė, Angelo Iovane, Antonino Bianco, Giuseppe Messina

**Affiliations:** 1 Sport and Exercise Sciences Research Unit, Department of Psychological, Pedagogical and Educational Sciences, University of Palermo, 90128 Palermo, Italy; cesanelli.leonardo@gmail.com (L.C.); angelo.iovane@unipa.it (A.I.); antonino.bianco@unipa.it (A.B.); 2 Ariminum Research & Dental Education Center, ARDEC Academy, 47923 Rimini, Italy; giancesa55@gmail.com; 3 Faculty of Sport Biomedicine, Institute of Sport Science and Innovations, Lithuanian Sports University, 44221 Kaunas, Lithuania; berta.ylaite@gmail.com

**Keywords:** sport performance, occlusion, mouthguards

## Abstract

The role of the dento-mandibular apparatus and, in particular, occlusion and jaw position, received increased attention during last years. In the present study, we aimed to systematically review, on the light of the new potential insights, the published literature covering the occlusal splint (OS) applications, and its impact on exercise performance. A structured search was carried out including MEDLINE^®^/PubMed and Scopus databases with additional integration from external sources, between March and June 2021. To meet the inclusion criteria, studies published in the English language, involving humans in vivo, published from 2000 to 2021 and that investigated the role of occlusal splints on athletes’ performance were selected. Starting from the 587 identified records, 17 items were finally included for the review. Four main aspects were considered and analyzed: (1) occlusal splint characteristics and occlusion experimental conditions, (2) jump performance, (3) maximal and explosive strength, and (4) exercise technique and biomechanics. The results of the systematic literature analysis depicted a wide heterogenicity in the experimental conditions and suggested the application of the OS as a way to improve athletes’ or individuals’ oral health, and as a potential tool to optimize marginal aspects of exercise performance.

## 1. Introduction

**Figure 1:**
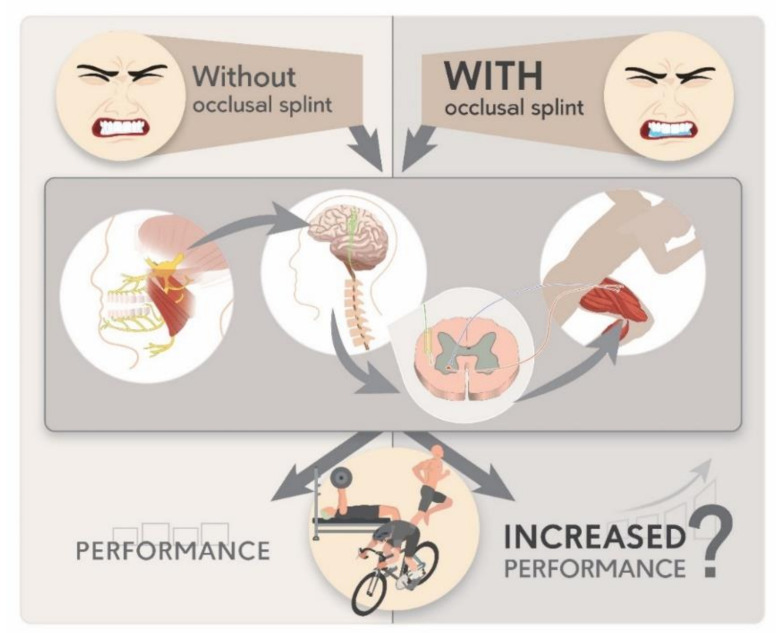
Graphical representation of how changes in dental occlusion (i.e., occlusal splint application vs. normal occlusion) may potentially affect human performance, through output signals transmitted in the trigeminal nerve, moved to the central nervous system, and in turn transferred it to the entire body system via spinal and autonomic nerves.

Athletic performance is the result of the joint interaction of a multitude of factors [1,2]. In-field and laboratory research, as well as clinical experience, led to the observation of the frequent symptomatic connections between the constitutive elements of the postural chain, and on how alterations of these connections may influence athletic performance through postural adaptations [3,4,5,6,7]. Thus, different interventions have been performed in order to analyze, and better understand the impact of postural chain connection alterations, on human performance [8,9]. Among them, the role of the dento-mandibular apparatus and, in particular, occlusion and jaw position, received increased attention, and became the object of several investigations published in recent years [5,9,10]. Indeed, studies have even reported that acute interventions in occlusion condition may induce positive effects in posture, postural control, balance, walking and running biomechanics, endurance, and strength performance [11,12,13,14,15,16,17]. In this regard, the application of different mandibular orthopedic repositioning appliance (MORA) devices, as the occlusal splints (OS), in order to enhance or re-establish athletes’ performance level, represented object of increasing interest in the scientific community. The principle behind the application of MORA, and in particular of the OS, is based on the observations by which dental occlusion (i.e., dynamic relationship between the maxillary and mandibular teeth when they approach each other) may affect physical performance by improvements of the temporomandibular joint (TMJ) alignment in the vertical dimension of occlusion, which under remote voluntary contraction (RVC) of the temporomandibular system (TMS) muscles (jaw clenching), may contribute to the phenomenon called concurrent activation potentiation (CAP) [4,18,19]. The neuromuscular effects of jaw repositioning and contraction of the TMS muscles may translate to improved neuromuscular responses in active exercise movers and consequently contribute to strengthen movements [4,15]. When a part of the motor cortex is active, connections to the other areas of the motor cortex are also affected [19]. Moreover, improvements in neuromuscular connections with jaw repositioning have been observed in muscular activation and proprioceptive feedbacks from the TMJ, projected via afferent fibers to accessory nerve nuclei, and influencing the extent of the neuromuscular response [4,15,18]. The current understanding is that the TMS and the whole-body neuromuscular system are connected via the central nervous system (CNS), and that changes in dental occlusion may affect both, static, dynamic muscle balance, and body posture, through output signals transmitted in the trigeminal nerve, which is associated to the mandibular proprioception [20,21]. This altered signal is then transmitted to the CNS, which in turn transfer it to the entire body system via spinal and autonomic nerves (Figure 1) [20,21,22]. Possible mechanisms of CAP include thus, increases in alpha motoneuron activity, gamma loops, and muscle spindles together with descending cortical input or stimulus-invoked afferent input [19]. This was observed through human, in vivo electromyography studies on muscle activation patterns in different occlusal conditions and by animal studies reporting linkages between neurons of the cranio-mandibular system and structures of the central nervous system [22,23,24,25]. In addition, indirect evidence suggested an additional hypothesis, by which the TMS and the skeletal muscles are connected with the fascia system through muscle and fascia chains, respectively, and how this may explain the neuronal connection of TMS and body posture [26]. Although several hypotheses and observations exist, the results of the few literature reviews that explored the possible associations between occlusion, jaw position, and athletes’ performance, brought out the complexity of this topic, underlying the presence of controversial results, and difficulties in testing procedures standardization [9,10,27]. However, in recent years the sciences applied to sport performance analysis as well as the knowledge associated with the dento-mandibular apparatus have progressed consistently, and this is demonstrated by the number of works covering this field, published in recent years. Thus, the aim of the present study is to systematically review, on the light of the new potential insights, the published literature on occlusal splint applications and athletes’ performance.

## 2. Materials and Methods

A computerized literature search was performed (March 2021 to June 2021) through two online databases (MEDLINE^®^/PubMed and Scopus), chosen to consider the major journals regarding the field of sports dentistry and exercise performance. Search terms included keywords addressing: “Dental Occlusion” [MeSH term] AND (“Splint” [MeSH term] OR “Bite” OR “MORA” OR “Occlusal Splint” [MeSH term] OR “Occlusal Bite”) AND (“Sport” [MeSH term] OR “Performance” OR “Athletic Performance” [MeSH term] “Exercise” OR [MeSH term] “Posture” [MeSH term] OR “Balance” OR “Postural Balance” [MeSH term] OR “Strength” OR “Muscle Strength” [MeSH term] OR “Endurance” OR “Physical Endurance” [MeSH term] OR “Walking” OR [MeSH term] “Running” [MeSH term]) filtered by “English language”, “last 20 years” and “Humans”.

**Figure 2:**
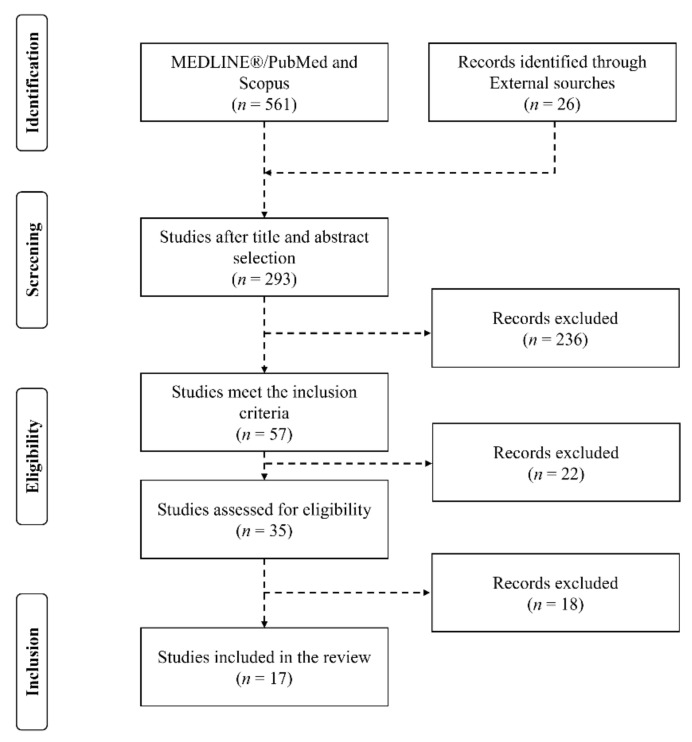
PRISMA flow-diagram.

All titles and abstracts from the search were cross-referenced to identify duplicates and any potential missing studies. Titles and abstracts were then screened for a subsequent full-text review. The search for published studies was independently performed by four authors (L.C., B.Y., G.C., and G.M.) and disagreements were resolved through discussion. The search results were downloaded and filtered in EndNote software (X8; Clarivate Analytics, New York, NY, USA). The search was concluded on 4 June 2021. The eligibility criteria were selected according to the PICO (Population, Interventions, Comparators, Outcomes) strategy as represented in Table 1 [28]. To meet the inclusion criteria, studies published in the English language, investigating humans in vivo, published from 2000 to 2021 and that investigated the role of occlusal splints on athletes’ performance were selected. Studies conducted on children (<12 years old), on untrained individuals, on disabled people, on use of supplements and/or drugs or clinical studies on diseased individuals have been excluded. Observational studies, controlled clinical trials (CCTs), and random controlled trials (RCTs) with randomization at any level have been selected for screening procedures (Figure 2).

Following the removal of duplicate studies from the different search engines, inclusion or exclusion of the remaining articles were applied following the aforementioned criteria on the title and abstract to determine eligibility in a preliminary independent screening. Selected papers were then read in full to analyze eligibility or exclusion. A summary of this process is outlined in Figure 2 [29]. A standardized Microsoft Excel spreadsheet have been used to extract and collate data, including study design and aim, characteristics of participants, investigation methods, and representativeness of the study sample and results (Table 2).

## 3. Results

A total of 17 studies met the inclusion criteria and were included in the current systematic review [4,5,15,18,22,23,30,31,32,33,34,35,36,37,38,39,40]. The predefined search strategy yielded a preliminary pool of 587 possible papers, screened following the steps described by the PRISMA model, and with the protocol approved by all the authors before launching the predefined search (Figure 2) [29]. Although the studies of Abdallah et al. (2004), Chakfa et al. (2002), Grosdent et al. (2014), Mitsuyama, Takahashi and Ueno (2016), and Zupan et al. (2018) involved exercise testing with interventions meeting the inclusion criteria of the present review, we excluded these works according to the physical activity level or characteristics of the investigated population [41,42,43,44,45]. Similarly, the study of Leroux et al. (2018), although of interest for the research question of our work, has been excluded considering the experimental design, in which, the occlusal splint was applied to induce occlusal disturbance rather than improve the vertical dimension of occlusion (VDO) [46]. The exercise testing involved in the included studies covered multiple aspects of physical performance ranging from aerobic to anaerobic capacity, maximal, explosive and endurance power, force, and strength, and through the evaluation of different markers (Table 2). The results of the present search have been thus discussed following distinct sections regarding the different exercise performance aspects considered by the included studies.

## 4. Discussion

### 4.1. Occlusal Splint Characteristics and Occlusion Experimental Conditions

The analysis of the OS devices’ characteristics revealed two main broad categories in which the OS can be grouped: (1) custom-made [4,5,15,18,22,23,31,32,33,34,35,36,37,38,39,40] and (2) commercial products [30,31,32,33,39,40] (Table 2). If for the commercial OS the personalization is limited (e.g., boil and bite OS) [38] or not possible (e.g., self-fit OS) [30], the custom-made splints can be tailored specifically on subjects’ mouth characteristics through dental impression stone or wax models (see, e.g., in [15,23]), semi-adjustable articulators (see, e.g., in [39]), or by digital reading models (see, e.g., in [18]). Five studies compared the impact of custom-made vs. commercial OS [31,32,33,39,40]. Both, Drum and colleagues [31] and Schulze, Kwast, and Busse [40] observed no significant differences in athletes wearing custom-made or commercial OS, both in terms of performance and in terms of comfort. Duddy et al. [32] reported too no differences in terms of performance, however, boil-and-bite commercial OS determined breathing difficulties, resulting uncomfortable for the subjects. Differently from the previously mentioned studies, Dunn-Lewis and colleagues [33] reported improved performance markers in custom-made OS conditions compared to commercial or no splint application. The experimental conditions in which the OS were applied, differed according to the jaw position: 64.7% upper jaw [4,18,22,30,31,32,33,34,38,39,40], and 35.3% lower jaw [5,15,22,34,35,36] (Table 2). It was possible to observe how 83.3% of the studies in which the OS was positioned in the lower jaw reported a significant improvement in at least one of the analyzed performance markers [5,15,23,35,37], while on the other side, 54.5% of the studies with the OS positioned in the lower jaw reported a significant improvement in one of the investigated parameters [4,18,32,38,39,40]. However, no study compared the possible impact of lower or upper jaw OS applications. In addition, we observed how in all the studies in which the OS has been positioned in the lower jaw the splints were custom-made [5,15,23,35,36,37], while for upper jaw OS it has been used both commercial [30,31,32,33,39,40], and custom-made splints [4,18,22,31,32,33,39,40], with different studies applying, and comparing the two categories of OS [31,32,33,39,40]. Taken together, from the present systematic analysis emerged the wide heterogenicity of the experimental conditions in which the impact of the OS has been investigated, including both intrinsic characteristics of the splints and its anatomical applications. These differences may potentially represent an explanatory factor behind the discordant results and observations made by different authors.

### 4.2. Jump Performance

Jump performance evaluation represented the most tested exercise with four studies involving the vertical squat jump (VSJ) test [23,33,37,38] and eight studies the counter movement jump (CMVJ) test [18,23,30,35,36,37,38,40]. The wide application of vertical jump testing such as the VSJ and CMVJ confirm it as one of the most frequently used tools to assess lower limb explosive strength [47]. The potential action of the CAP effect induced by the jaw clenching (RVC) and the application of the OS, in a static (VSJ) and dynamic (CMVJ) exercise performance was thus object of different investigations. Four studies found significant improvements in jump performance following the application of OS for both VSJ and CMVJ [18,23,35,37] while five studies observed no significant differences [30,31,33,36,40]. As the effectiveness of RCVs potentiation phenomenon has been demonstrated for isometric and static actions, while less in dynamic actions, it was interesting to notice the greater application of the CMVJ compared to the VSJ in the experimental design of the included studies [19,48]. This can be explained by the insights given by testing a jumping condition where a reflector pathway is involved as the CMVJ, leading to the observation of the possible interactions between the reflector pathway and the modulated state of the premotor cortex due to changed afferent signals [4]. The possible ergogenic effects induced by the OS, leading to improvements in jump performance, is probably the result of a combination of several factors as underlined by different authors [18,23,33,35,37]. The OS have in common that they adjust the condyle in a more centric position, which decompresses the jaw joints on both side of the body. Jaw clenching with the protective role of the mouthpiece, the better comfortability, and the optimized occlusion resulting from the reposition of the TMJ in a centric relation position by the OS, allow a potentially increased occlusal stability of the jaw through bilateral simultaneous and symmetrical contacts of the teeth in both TMJs. This should lead to a relaxation of the jaw muscles and consequently to a balanced occlusion in terms of balanced occlusal contact points. Additionally, a compression of the jaw joints can be avoided while biting. Together these factors may induce changes in the temporo-mandibular system. Moreover, those neuromuscular changes may be transmitted to the whole body via neural connections as well as active and passive tissues (e.g., muscles and fascia). The change of the jaw position may alter thus the sensory signals to the brain. The increased stability leads also to an upsurge in EMG activity in the masseter muscles during strength or power tests due to an involuntary contraction of the mandible [4,23]. Moreover, changes in proprioceptive feedback may occur, which project information via afferent fibers from the masticatory system to the accessory nerve nucleus that controls the sternocleidomastoid and upper limb muscles like the trapezius [4]. It is well known that transcortical and subcortical pathways exist which can change motor output based on alterations in sensory information [49,50,51,52]. This may also be the case for interactions between jaw position and motor output. It was also previously demonstrated how voluntary jaw clenching could facilitate H-reflexes’ of muscles of different body areas (e.g., forearms) as a consequence of the CAP effect [53], potentially explaining also the differences observed in handgrip PF [15,23]. Haughey and Fine [35] described how additionally, the application of the OS may create more free space causing TMJ decompression and allowing the tongue to posture in a more anterior position increasing upper airway space. As a consequence, this may change cervical spine curvature by reducing forward head posture [35], influencing function and structure throughout the muscle chains and explaining how a lower jaw position can influence musculatures not directly corrected to the lower jaw and exercise performances involving lower limb muscles as the jumps [35]. However, it remains unclear whether the change in sensory condition-introduced by the application of the OS and the consequent changed jaw position results in the observed change of the jump exercise performance and motor output, or whether a different mechanism is responsible.

### 4.3. Maximal and Explosive Strength

The impact of the OS application on the ability to peak force by a muscle or muscle group, has been evaluated by different authors through several exercise tests. Three studies included the 1RM test by choosing a bench press exercise [30,33] or the leg press exercise [37]. Three studies analyzed the impact on the peak hand-grip force (PF) [5,18,23] while Buscà et al. [18] and Dias et al. [4] included, respectively, an isometric PF and isokinetic strength evaluation. Early studies performed at the end of the 20th century suggested the inefficacy of OS in improving muscular strength [54,55,56]. This have been further confirmed by part of the more recent studies included in this review with no effect on 1RM test for the bench press [30,33]. However, Maurer et al. [37] observed improvements (+3 to 12%) in both, maximal (peak force) and explosive (RFD) strength, by the application of OS in the leg press exercise. Dunn-Lewis et al. [33] found significant improvements in the vertical jump RPD as well as improved peak power output and peak force in a supine plyo press exercise and improved bench throw peak force and power, using the OS. On the contrary, Allen and colleagues [30] found non-significant slight improvements in CMVJ peak force and RFD. Buscà et al. [18] observed an improvement isometric back row exercise PF by the application of the OS. Dias et al. [4] described positive ergogenic effects on shoulder muscular strength through the application of the OS, observing improved isokinetic strength and EMG activity for some of the tested muscles. The already mentioned, H-reflexes’ facilitation of the forearm muscles during a voluntary jaw clenching observed by Takahashi, Ueno, and Ohyama [53] and the CAP effect could explain the differences in handgrip PF reported by the studies of Buscà et al. [18] and Carbonari et al. [23]. It has been previously described how different studies also evaluated the possible impact of OS on explosive strength, defined as the ability to exert maximal force in the shortest time interval [30,31,32,34,37,39,41]. In addition, several studies also evaluated the impact of OS on sprint and anaerobic performance [32,33,34,38,40] and on reaction time [31,33]. Non-significant differences have been observed by the three studies [32,33,40] that evaluated the impact of the OS on sprint performance while discordant results emerged from the study of Fischer, Weber, and Beneke [34] that reported no impact of the OS on Wingate test results, and the study of Morales et al. [38] in which was observed significant positive effects on anaerobic performance. Taken together, there are evidence supporting the possible ergogenic effect of the application of the OS in improving muscular strength, however the lack in experimental conditions standardization, the presence of controverse results and a lack of full understanding of the possible pathways leading to such improvements make it still difficult to claim as ergogenic or ineffective.

### 4.4. Exercise Technique and Biomechanics

Three studies focused on different aspects as running kinematics [15], precision, and exercise technique [22,39]. Previous studies reported how small interventions at the jaw may have significant effects on posture and gait stability [12,13] and we already mentioned the hypothesis by which the TMS and the neuromuscular system of the whole body are connected via the CNS, with afferent pathways from the TMS connected with efferent neurons affecting body posture [24,25,26]. Thus, Maurer et al. [15] hypothesized an impact not only on standing and walking stability but investigated the impact of the OS application on running kinematics. The authors observed singular and subjective adaptative patterns that anyway demonstrated to be in a more symmetrical running when compared to neutral conditions. Dias et al. [12] observed no significant differences in body posture, upper limb EMG and shot performance of 10-m pistol shooters using occlusal splints or placebo splints while Pae and colleagues [39] observed improved performance (greater club head speed and driving distance) in professional golfers. Additional studies are necessary to evaluate the possible impact of the OS on biomechanical aspects, exercise motion and technique.

## 5. Conclusions

The present review underlined the gradual and continuous increase of interest in research applied to the OS and its implications in sport and exercise science. This, in parallel with the improvements in exercise performance analysis methodologies and tools, is allowing a deeper understanding of the possible impacts of the OS on sport and exercise. However, different open question remains unresolved, and this is accompanied by a lack of homogeneity in research design, research questions, and applied methodologies, including intrinsic characteristics of the splints and its anatomical applications, to deeper investigate the present topic. To date, due to the still conflicting reports, it is thus difficult to claim that the application of the OS can impact consistently on exercise performance. However, data are promising, especially in exercises in which the CAP effect induced by a proper jaw clenching (RVC) and supported by the application of OS, can have an impact. The application of the OS can be thus viewed first as a way to improve athletes’ or individuals’ oral, but not only, health, and a possible tool to optimize marginal aspects of exercise performance. Medical staff, coaches, and all the practitioners involved in exercise performance optimization may thus focus first on the primary determinants of exercise performance and, after considering subjective characteristics of the athlete, work on secondary aspects as the OS can be considered.

## Figures and Tables

**Table 1:** Summary of the inclusion criteria following the PICO approach.

Parameter	Inclusion Criteria
Participants	Physically active and/or trained individuals without TMJ dysfunctions
Intervention	Application of occlusal splints in order to evaluate the impact on exercise performance
Comparison	Any
Outcome	Analysis of occlusal splints impact on exercise performance

**Table 2:** Data extraction of the included studies.

Year	Authors	Population	Intervention	Outcomes	OS Category	OS Position
2009	Manfredi et al. [36]	15 M élite basketball players	CMVJ; unilateral and bilateral stiffness jump	Performance tests: ~^ns^	Cus	LJ
2012	Duddy et al. [32]	18 M rowers	3-stroke maximum power test; 1-min ergometer tests; 1600-m run.	3-stroke maximum power test: ↑ *; 1-min ergometer tests: ↑ ^ns^; 1600-m run: ~^ns^.	Cus + Com	UJ
2012	Dunn-lewis et al. [33]	26 M trained and 24 F trained	Sit-and-reach FLEX; medial-lateral balance; visual RT; VJ; 10-m sprint; bench throw; plyo press power quotient	Sit-and-reach FLEX: ↑ ^ns^; medial-lateral balance: ~^ns^; visual RT: ↑ ^ns^; VJ: ↑ ^ns^; VJ RPD: ↑ * (M only); 10-m sprint: ↓ ^ns^; bench throw: ↑ *; plyo press power quotient: ↑ *(M only)	Com	UJ
2012	Pae et al. [39]	8 professional golfers	10 drive swings and 10 putts	Driving distance: ↑ *; club head speed: ↑ *	Cus + Com	UJ
2014	Allen et al. [30]	21 M physically active	CMVJ; 1RM bench press	CMVJ: ↓ ^ns^; PF: ↑ ^ns^;RTD: ↑ ^ns^; 1RM: ↑ ^ns^	Com	UJ
2015	Buscà et al. [18]	28 M physically active	CMVJ; Handgrip PF; Isometric back-row PF	Handgrip PF: ↑ *; CMVJ: ↑ *; Isometric back-row PF: ↑ *	Cus	UJ
2015	Maurer et al. [15]	20 M recreational runners	3D kinematic analysis of running	Running symmetry: ↑ *	Cus	LJ
2015	Morales et al. [38]	28 M physically active	Wingate test 30 s; Spirometry	Wingate test parameters: ↑ *; [La-]b: ↓ *; Vemax: ↑ *	Cus	UJ
2016	Drum et al. [31]	10 M NCAA division II football players	RT; [La^-^]_b_; FLX; CMVJ; VJ; 1RM bench press.	RT: ↓ ^ns^; [La^-^]_b:_ ↑ ^ns^; FLX: ↑ ^ns^; CMVJ: ↓ ^ns^; VJ: ↓ ^ns^; 1RM bench press:↑ ^ns^.	Cus + Com	UJ
2016	Fischer, Weber and Beneke [34]	23 M physically active	Wingate test 30 s	Wingate test parameters: ~^ns^	Cus	UJ
2018	Battaglia et al. [5]	25 M martial arts athletes	Handgrip PF	PF: ↑ *	Cus	LJ
2018	Dias et al. [22]	13 M national level shooters	Shooting score	Shooting score: ↓ ^ns^	Cus	UJ
2018	Maurer et al. [37]	23 M recreational runners	VJ; CMVJ; DJ; trunk flexion and extension; leg press.	VJ: ↑ *; CMVJ: ↑ *; DJ: ↑ *; trunk flexion and extension: ↑ *; leg press: ↑ *; Leg press RFD: ↑ *.	Cus	LJ
2019	Dias et al. [4]	14 M healthy subjects	Shoulder abduction/adduction and arm external/internal rotation isokinetic strength, (concentric 60°/s)	Abduction: ↑ *; Adduction: ↑ *; Internal rotation: ↑ *; External rotation: ↑ *	Cus	UJ
2019	Schulze et al. [40]	13 M first league rugby players	Lungs function test; incremental treadmill test; 20 m and 40 m sprints; CMVJ	PEF: ↓ *; Performance parameters: ~^ns^	Cus + Com	UJ
2020	Carbonari et al. [23]	18 well-trained subjects (11 M; 7 F)	VJ; CMVJ; Handgrip PF	Handgrip PF: ↑ *; VJ: ↑ *; CMVJ: ↑ *	Cus	LJ
2020	Haughey and Fine [35]	15 M athletes (gaelic football, field hockey and boxing)	CMVJ; Seated medicine ball (9 kg) putt; seat-and-reach test; passive knee flexion; balance test.	CMVJ: ↑ *; Seated medicine ball (9 kg) putt: ↑ *; seat-and-reach test: ↑ *; passive knee flexion: ↑ *; balance test: ↑ *.	Cus	LJ

M: males; F: females; ↑: increase; ↓: decrease; ~: unchanged; *: statistically significant increase/decrease; ^ns^: non-significant increase/decrease; CMVJ: countermovement vertical jump; VSJ: vertical squat jump; 1RM: one-repetition maximum test; PF: peak force; RTD, RFD, RPD: rate of torque/force/power development; RT: reaction time; [La^-^]_b_: blood lactate concentration; FLX: flexibility; Cus: Custom-made; Com: Commercia; LJ: lower-jaw; UJ: upper-jaw.
